# Plant diversity and root traits benefit physical properties key to soil function in grasslands

**DOI:** 10.1111/ele.12652

**Published:** 2016-07-26

**Authors:** Iain J. Gould, John N. Quinton, Alexandra Weigelt, Gerlinde B. De Deyn, Richard D. Bardgett

**Affiliations:** ^1^ Lancaster Environment CentreLancaster UniversityBailriggLancasterLA1 4YQUK; ^2^ Department of Special Botany and Functional BiodiversityInstitute of BiologyUniversity of Leipzig04103LeipzigGermany; ^3^ German Centre for Integrative Biodiversity Research (iDiv) Halle‐Jena‐Leipzig04103LeipzigGermany; ^4^ Department of Soil QualityUniversity of WageningenDroevendaalsesteeg 46708PBWageningenThe Netherlands; ^5^ Faculty of Life SciencesThe University of ManchesterMichael Smith Building, Oxford RoadManchesterM13 9PTUK

**Keywords:** Aggregate stability, biodiversity, grasslands, root traits, soil physics

## Abstract

Plant diversity loss impairs ecosystem functioning, including important effects on soil. Most studies that have explored plant diversity effects belowground, however, have largely focused on biological processes. As such, our understanding of how plant diversity impacts the soil physical environment remains limited, despite the fundamental role soil physical structure plays in ensuring soil function and ecosystem service provision. Here, in both a glasshouse and a long‐term field study, we show that high plant diversity in grassland systems increases soil aggregate stability, a vital structural property of soil, and that root traits play a major role in determining diversity effects. We also reveal that the presence of particular plant species within mixed communities affects an even wider range of soil physical processes, including hydrology and soil strength regimes. Our results indicate that alongside well‐documented effects on ecosystem functioning, plant diversity and root traits also benefit essential soil physical properties.

## Introduction

Rapid declines in biodiversity present a major threat to ecosystem functioning, and are considered a principal driver of environmental change (Hooper *et al*. 2005; Cardinale *et al*. 2012). Over the past few decades, such concerns have directed research towards gaining an understanding of how biodiversity loss influences ecosystem functioning and services. Within this context, many biodiversity‐ecosystem functioning studies have tested how plant diversity loss impacts multiple ecosystem functions and services (Isbell *et al*. 2011; Cardinale *et al*. 2012; Allan *et al*. 2013). While many of these studies have explored belowground effects of plant diversity loss, they have largely focussed on biological properties and processes, such as microbial community composition and activity, decomposition and the recycling of carbon and biologically essential nutrients (Scherer‐Lorenzen *et al*. 2003; Eisenhauer *et al*. 2010; Lange *et al*. 2015); as a result, little is known of the impact of plant species loss on the soil physical environment. This represents a significant gap in our knowledge of biodiversity‐ecosystem function relationships given the fundamental importance of soil physical structure to soil function, and its capacity to sustainably deliver ecosystem services (Amundson *et al*. 2015; Keesstra 2016). Further, as stated in the UN's ‘Status of the World's Soil Resources’ report, one‐third of the earth's soils are suffering from degradation, and much of this is due to declines in the physical structure of soil (FAO 2015).

Many factors influence soil physical structure, including texture and mineralogy, climate, organic matter supply from manures and crop residues, and disturbance from tillage, grazing animals and heavy machinery (Six *et al*. 2004). However, another important factor is the belowground activity of plants, and specifically the functional traits of roots, which impact the soil physical environment via a range of mechanisms (Bardgett *et al*. 2014; Schroeder‐Georgi *et al*. 2016). For instance, architectural root traits, such as root length, directly impact soil structure through binding and compressing soil particles, which influences soil aggregation and aggregate stability (Miller & Jastrow 1990; Six *et al*. 2004; Gyssels *et al*. 2005). In addition, root architecture can contribute to soil hydraulic conductivity and capacity to reduce run off, forming hydraulically effective pathways for water flow through soils; and can increase the root reinforcement effect on soil shear strength, promoting slope stability and erosion control (De Baets *et al*. 2007; Stokes *et al*. 2009; Macleod *et al*. 2013). Physiological root traits, especially root exudation, impact on soil structure by stimulating microbial activity and through the production of polysaccharides and proteins (Oades & Waters 1991; Kuzyakov 2010; Graf & Frei 2013). These exudates can also stimulate mycorrhizal fungi, whose hyphae extend into the soil matrix, acting as an extension of the root system to aid plant nutrient uptake while also increasing aggregate stabilisation (Leifheit *et al*. 2013). This effect is due to the physical enmeshing of soil particles by extensive mycorrhizal networks (Hallett *et al*. 2009), as well as the role of glomalin‐related protein, which forms hydrophobic surfaces providing a ‘glueing’ effect, bonding particles together (Wu *et al*. 2014; Rillig *et al*. 2015). While the impact of plant diversity on ecosystem processes and services has been found to be strongly driven by the traits of particular species within a plant community (Diaz *et al*. 2004; de Bello *et al*. 2010; Lavorel & Grigulis 2012), including the traits of roots (Mueller *et al*. 2013; Legay *et al*. 2014), we are yet to fully understand the role of root traits in explaining diversity effects on the physical environment belowground (Bardgett *et al*. 2014).

Although a few studies have reported broad relationships between plant species richness and soil physical properties (Pohl *et al*. 2009; Pérès *et al*. 2013; Berendse *et al*. 2015), remarkably little is known about how variation in plant diversity and composition impacts physical properties of soil. Moreover, the mechanisms involved remain unresolved, especially regarding the role of root traits with known potential to impact soil physical properties via a range of mechanisms (Bardgett *et al*. 2014). Using a subset of plots (1, 4 and 16 species) from the Jena grassland diversity field experiment, for example, Pérès *et al*. (2013) detected a positive effect of higher plant diversity on aggregate stability, but the role of root traits, apart from root biomass, was not explored. Also, in an experiment including four diversity treatments (1, 2, 4 and 8 species), Berendse *et al*. (2015) found that loss of plant species diversity reduces soil erosion resistance on slopes, but the physical mechanisms that control soil erodibility were not explored. Here, we combined a mechanistic mesocosm experiment with sampling of soils from the long‐term Jena grassland diversity experiment (Roscher *et al*. 2004) to investigate how plant diversity and community composition influence a comprehensive range of soil physical properties in grassland, and tested the hypothesis that diversity effects are explained by root functional traits with known potential to impact soil structure. Specifically, the mesocosm experiment enabled us to disentangle the impact of plant species richness and identity on soil physical properties, and identify the role of specific root traits; whereas the field experiment allowed us to test whether plant diversity effects on soil physical properties observed in our experimental studies could also be detected and explained by the same root traits across a wide gradient of species richness in the field, 10 years after the experiment was established. To the best of our knowledge, our study offers the first substantive experimental exploration of the mechanistic links between plant diversity and the soil physical environment at different experimental scales, and of the role of root traits with known potential to impact structural properties of soil. Moreover, we include in our analyses a range of soil physical properties of fundamental importance to soil function, including different measures of soil aggregate stability, soil saturated hydraulic conductivity to assess water flow regimes through the medium and a novel approach of *in situ* shear resistance testing in mesocosms to measure root reinforcement of soil strength. As such, our analysis provides a comprehensive assessment of the impact of root traits across plant diversity gradients on the soil physical environment over short and longer timescale.

## Materials and methods

### Mesocosm experiment

Mesocosm plant communities were established in two paired sets of 64 mesocosms using a substitutive planting design, devised by De Deyn *et al*. (2009). Two identical sets of mesocosms were required on account of the destructive nature of soil shear strength testing, which would not allow accurate sampling of aggregates or roots. This involved growing species from a pool of six common grassland species including grasses, legumes and forbs, covering a spectrum of root architectural traits (Cope & Gray 2009), for 18 months in glasshouses. Initial planting density was kept constant, while plant species and functional group richness varied from monocultures to six‐species mixtures. The plant species used were: grasses, *Lolium perenne* and *Anthoxanthum odoratum;* forbs, *Plantago lanceolata* and *Achillea millefolium;* and legumes, *Trifolium repens* and *Lotus corniculatus*, in addition to a bare soil treatment. The substitutive planting design kept initial plant density in terms of number of individuals per surface area constant for each community, while allowing equal representation in terms of frequency of presence of each species and functional group across blocks and species richness levels. Each pot was planted with 24 plant individuals, and the composition of each community depended on the assigned treatment. The experiment was a random block design, using four separate glasshouses, each acting as a block. Within each block, there were 16 different plant community assemblages, and two duplicate mesocosms for each: one for aggregate/root analysis and the other for hydrology/strength analysis, totalling 32 mesocosms per glasshouse. The position of each treatment combination per block was randomly allocated.

Each mesocosm consisted of a cylindrical pot of 50 cm depth and 30 cm diameter. For the 64 hydrology/strength‐testing mesocosms, a pre‐determined shear plane was cut in each pot at 8 cm soil depth (see supplementary methods), before securing both sides back together. The shear plane was protected with a PVC inner lining in each mesocosm to prevent disturbance from water or plant roots during the growing period. At the base was laid 10 cm of limestone chippings, above which was packed 35 cm of experimental soil at 1.3 g cm^−3^ density. The soil used was a Kettering Loam (clay loam, pH 7.4, C_org_ of 45 g kg^−1^ and N concentration of 2.1 g kg^−1^) provided by Boughton Loam Ltd. (Kettering, UK). The soil was unsterilised and had been screened to 3 mm prior to packing. Plants were grown from seed in controlled environment rooms (16 h day length, 24 °C day temp., 16 °C night temp.) and subsequently planted into mesocosms in glasshouses at Hazelrigg Field Station, Lancaster University, UK (54°1′N, 2°46′W). Mesocosms were irrigated via an automated sprinkler system, and aboveground biomass was cut twice yearly.

At final harvest, aboveground biomass of each individual species was measured after drying at 70 °C for 48 h. For root trait analysis, three soil cores of 3.2 cm diameter and 10 cm depth were sampled from each mesocosm pot. In one set of 64 mesocosms, aggregate stability was undertaken on air‐dried soil aggregates. The Le Bissonais (1996) method of aggregate stability measurement was used, which subjects the aggregates to three breakdown mechanisms representing different environmental pressures: slaking (rapid immersion in water for 10 min), microcracking (slow wetting on a −0.3 KPa tension table for 1 h) and mechanical breakdown (placing in a 250 mL conical flask and agitating end over end 10 times by hand). After each breakdown, aggregate samples were sieved in ethanol before oven drying to determine final aggregate size distribution, producing a mean weight diameter (MWD) (Le Bissonais 1996). Final aggregate size classes Quebec City following breakdown are reported in Supplementary Fig. S1. Root traits were measured using WinRHIZO (Regent Instrument, Quebec, Canada) followed by drying at 70 °C for 48 h. We then used these measurements to calculate root length density, root mass density, average root diameter, specific root length, root dry matter content and root tissue mass density. Soil organic matter was determined by the loss on ignition method. The second set of 64 mesocosms was contained in pots adapted for the measurement of (i) saturated hydraulic conductivity (*K*_sat_) and (ii) root reinforcement of soil strength. The falling head method of measuring *K*_sat_ was employed, utilising the mesocosm container as a permeameter to calculate the rate of water flow based on dimensions of the soil column and the fall in head over time (Klute & Dirksen 1986; Smith & Mullins 1991). For an assessment of root contributions to soil strength, a shearing rig was developed to fit complementary to the mesocosm pot (see Supplementary methods), applying a shear load to the exposed shear plane. A hydraulic ram applied a constant loading to the soil column, while force and displacement of the top half of the soil column were logged with a CR800 datalogger (Campbell Scientific Ltd, Leicestershire, United Kingdom). Data output from the shearing process was used to derive a value of root reinforcement of shear strength for each mesocosm. For further details of *K*_sat_ and strength testing, see supplementary methods information.

### Field experiment

The long‐term grassland biodiversity experiment at Jena, Germany (11°37′27″ E, 50°57′4″ N) was established in 2002 on the floodplain by the Saale River. Mean annual temperature was 9.9 °C with annual precipitation of 610 mm (Hoffman *et al*. 2014). The soil was an Eutric Fluvisol, which spatially varied in texture from sandy loam to silt loam; this variation was incorporated into the parallel block design of the experiment, each block encompassing a slight change in soil texture. Prior to sowing, the soils had a pH range of 7.1–8.4, C_org_ of 5–33 g kg^−1^ and N concentration of 1.0–2.7 g kg^−1^ (Roscher *et al*. 2004). The experiment comprised 82 plots of 20 × 20 m, with manipulated species richness of 1, 2, 4, 8, 16 and 60. Plant species were selected from a pool of 60 European grassland species, from four functional groups: grasses, legumes, tall herbs and short herbs (Roscher *et al*. 2004). With the exception of *L. perenne*, all of the plant species used in the mesocosm experiment were included in the field experiment. The experiment was set up in spring 2002, and sampling was undertaken in summer 2012. Within a 0.3 × 1.6 m strip per plot, two ~ 500 g topsoil samples were extracted with a trowel to 10 cm depth and bulked for soil analysis, and three 3.2 cm diameter cores were taken to 10 cm depth and bulked for root analysis. Aggregate stability and root trait analysis were conducted as in the mesocosm experiment, only with a reduced root trait suite (root length density and root mass density only). Soil samples were also used to determine organic matter content by loss on ignition and glomalin‐related protein content (Rosier *et al*. 2006; Koide & Peoples 2013).

### Data analysis

For all mesocosm analysis, we used general linear models (GLM) with block as a random effect and treatments as fixed effects. Prior to analysis of the mesocosm soils, data for aboveground biomass, root length density, root mass density, slaking aggregate stability, hydraulic conductivity and shear strength were log transformed in order to satisfy the assumptions of normality. The effects of species richness on soil physical properties and root traits were analysed using mixed effects linear models on all mesocosms. For species‐specific effects, using mesocosm data only, the effect of monoculture species on root traits, organic matter and aggregate stability was tested with GLM; differences between treatments were determined using Tukey's post hoc testing. Relationships between root traits, soil organic matter content and physical properties were investigated with linear regression, as were relationships between aboveground biomass (at final harvest) of each species and soil physical properties. Following the linear regression, based on collinearity and variance inflation factors (where VIF < 5), we selected root length density, root mass density, soil organic matter content and aboveground biomass, to fit before species richness in ancova analysis to investigate their effects on soil physical properties in the mesocosms using sequential type 1 sum of squares models, where species richness was always fitted last, and alternating the order of root traits fitted before. Reported *F* and *P* values indicate where respective factor was fitted first (Schmid *et al*. 2002). Monoculture data found one of the species, *L. perenne*, to exhibit a notably influential relationship with aggregate stability and root production; as such, we tested the role of *L. perenne* in plant species mixtures across the species richness gradient by repeating models with root length density as a factor and aggregate stability as a response, however, separating the data for communities with and communities without *L. perenne*.

For analysis of the field soils, we included block first in all models, in line with previous analyses of data from the Jena experiment (Roscher *et al*. 2005), and data for sown plant diversity were log transformed in order to meet the assumptions of normality. Block was included first in the field analysis to account for the slight gradient in soil properties across blocks (Roscher *et al*. 2004). manova and subsequent anova as part of a general linear model (GLM, type 1 sum of squares) was used to analyse the effects of block, plant species richness, plant functional group richness and the presence of each functional group on the different aggregate stability measures. We performed analyses in a hierarchical order, whereby species richness and functional group richness were fitted first, followed by an alternating order of functional group presence (grasses, legumes, tall herbs, short herbs). manova was employed as a multivariate analysis to assess the community effects on a number of dependant variables, in this case all three aggregate stability measures. anova (type 1 SS) was also employed to test the effects of the community composition on root length density, root mass density, organic matter content and glomalin‐related protein. Reported *F* and *P* values are those where respective factor was fitted first. Root length density, root mass density, organic matter content and glomalin‐related protein were then fitted before species richness, functional group richness, legumes and grass presence, in ancova analysis to investigate their effects on soil physical properties. All analyses were carried out in R.3.0.1 (R Development Core Team, 2013).

## Results

### Mesocosm experiment

**Figure 1:**
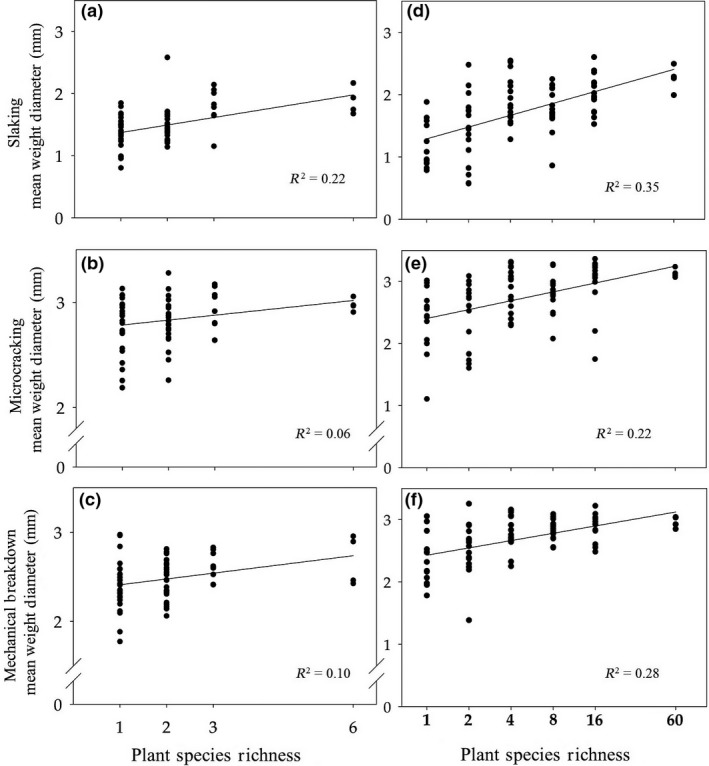
Plant Species richness impacts on soil aggregate stability in the mesocosm (a–c) and the Jena (d–f) field plots soils. All six regression lines indicate a significant relationship *P* < 0.05. *X*‐axis for the field soils (df) is log transformed.

Species richness had a positive effect on all three aggregate stability measures (Fig. 1a–c) (slaking: *F*_1,55 _= 15.65, *P* < 0.01, microcracking: *F*_1,55 _= 4.09, *P* < 0.05, mechanical breakdown: *F*_1,55 _= 6.69, *P* < 0.05). In addition, species richness had a positive impact on aboveground biomass (*F*_1,55 _= 6.17, *P* < 0.05), but had no effect on the measures of soil strength (*F*_1,45 _= 0.05, *P* > 0.05) or hydraulic conductivity (*F*_1,45 _= 3.75, *P* > 0.05).

Root traits varied among plant species (Table 1). The grasses *L. perenne* and *A. odoratum* displayed the greatest root length density, specific root length and narrowest average diameter. *Trifolium repens* had the lowest root length density, root mass and tissue density, and the highest dry matter content, while the other legume *L. corniculatus* had the greatest root mass, diameter and tissue density, and consequently the lowest specific root length. Plant diversity influenced a number of community root traits. Increased species richness caused greater root length density and lower average diameter and dry matter content (Supplementary Table S1). Nevertheless, root mass density, specific root length and tissue mass did not change as a consequence of species richness (Supplementary Table S1). Soil organic matter content did not vary between monocultures (*F*_6,18 _= 1.32, *P* = 0.30) and was not influenced by species richness (Supplementary Table S1).

**Table 1:** Variation in soil physical properties and root traits under monocultures in the mesocosm experiment

Soil physical properties	Species						
	Grasses		Legumes		Forbs		
	Lp	Ao	Tr	Lc	Am	Pl	Bare soil
Aggregate stability (mm)							
Slaking	1.61 ± 0.1^a^	1.47 ± 0.09^ab^	1.11 ± 0.15^b^	1.14 ± 0.09^ab^	1.35 ± 0.13^ab^	1.50 ± 0.11^ab^	0.71 ± 0.04^c^
Microcracking	2.77 ± 0.11^a^	2.92 ± 0.09^a^	2.69 ± 0.14^ab^	2.64 ± 0.2^ab^	2.66 ± 0.14^ab^	3.01 ± 0.03^a^	1.99 ± 0.35^b^
Mechanical breakdown	2.67 ± 0.11^a^	2.52 ± 0.03^a^	2.17 ± 0.13^ab^	2.15 ± 0.13^bc^	2.32 ± 0.04^bc^	2.59 ± 0.18^abc^	1.90 ± 0.15^c^
Hydrology (mm h^−1^)							
Saturated hydraulic conductivity	1.82 ± 0.74^ns^	8.69 ± 8.18^ns^	6.54 ± 2.63^ns^	23.05 ± 3.53^ns^	8.9 ± 6.1^ns^	3.97 ± 1.99^ns^	10.92 ± 6.53^ns^
Strength (kN m^−2^)							
Root reinforcement of soil strength	10.78 ± 3.66^b^	17.41 ± 7.16^b^	4.47 ± 2.24^b^	28.55 ± 5.39^a^	10.21 ± 3.45^b^	1.52 ± 1.38^b^	NA
Root Traits							
RLD (m dm^−3^)	689.8 ± 99.6^a^	568.6 ± 46.8^a^	135.9 ± 11.7^c^	230.9 ± 39.2^b^	193.5 ± 25.5^bc^	496.5 ± 33.5^a^	NA
RD (g dm^−3^)	4.03 ± 0.84^ab^	2.53 ± 0.19^bc^	1.05 ± 0.23^c^	5.79 ± 0.86^a^	2.51 ± 0.36^bc^	4.1 ± 0.56^ab^	NA
RDIAM (mm)	0.22 ± 0.009^d^	0.21 ± 0.009^d^	0.33 ± 0.02^b^	0.41 ± 0.01^a^	0.33 ± 0.02^b^	0.26 ± 0.005^c^	NA
SRL (m g^−1^)	177.0 ± 9.6^a^	231.7 ± 34.4^a^	149.7 ± 35.2^ab^	40.5 ± 5.7^c^	79.3 ± 8.7^b^	127.6 ± 14.8^ab^	NA
DMC	0.13 ± 0.003^b^	0.16 ± 0.008^ab^	0.19 ± 0.016^a^	0.17 ± 0.02^ab^	0.16 ± 0.01^ab^	0.14 ± 0.008^b^	NA
TMD (g cm^−3^)	0.15 ± 0.01^ab^	0.12 ± 0.006^bc^	0.08 ± 0.012^c^	0.19 ± 0.02^a^	0.15 ± 0.02^ab^	0.15 ± 0.01^ab^	NA

Mean values ± SE for soil physical properties and root traits from monocultures of *Lolium perenne* (Lp), *Anthoxanthum oderatum* (Ao), *Trifolium repens* (Tr), *Lotus corniculatus* (Lc), *Achillea millefolium* (Am), *Plantago lanceolata* (Pl) and bare soil. Root traits include root length density (RLD), root mass density (RD), root diameter (RDIAM), specific root length (SRL), dry matter content (DMC), tissue mass density (TMD). Letters indicate significant differences between plant treatments *P* < 0.05 by Tukey's post hoc testing.

Soils planted with monocultures of the grasses *L. perenne* and *A. odoratum* had greater aggregate stability against mechanical breakdown than did soils planted with forbs or the legume *L. corniculatus*, and soils planted with *L. perenne* exhibiting the greatest stability against slaking (Table 1). *L. corniculatus* had the greatest influence on soil strength when grown in monocultures (Table 1), and aboveground biomass of *L. corniculatus* was positively correlated with soil strength in mixed communities (*F*_1,14 _= 10.56, *P* < 0.01, *R* ^2 ^= 0.34). When grouped together according to plant functional type, soil planted with legumes displayed greater hydraulic conductivity than the other functional groups (legumes 19.76 ± 3.46 mm h^−1^; compared to grasses 8.24 ± 1.49 mm h^−1^, *P* < 0.005; compared to forbs 8.52 ± 3.52 mm h^−1^, *P* < 0.01).

**Figure 2:**
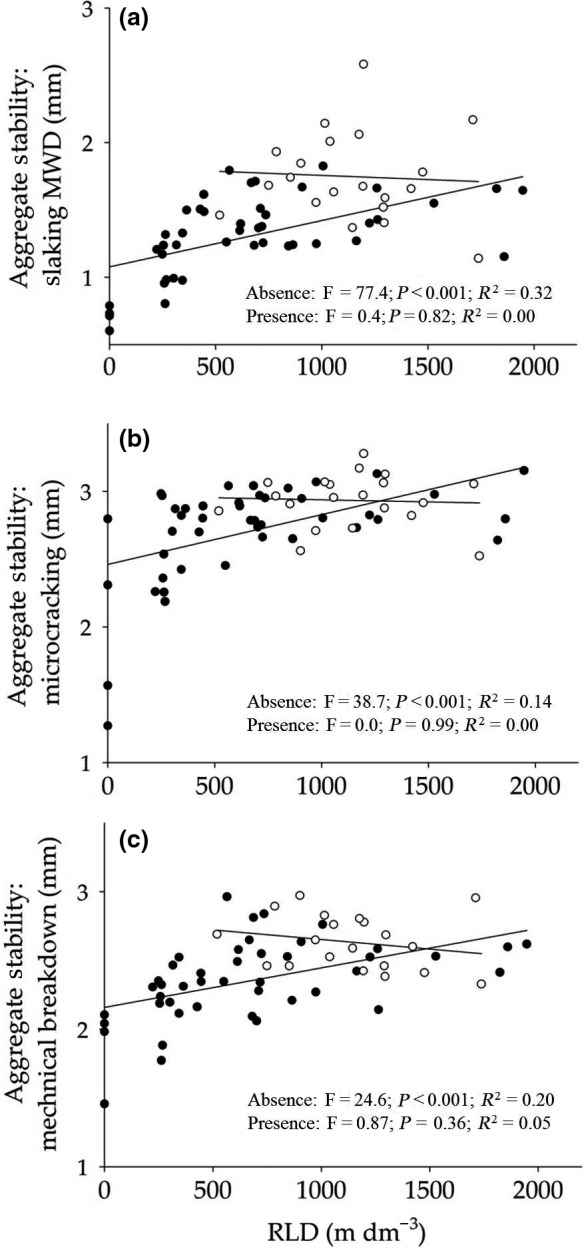
The influence of Lolium perenne on root length density effects on soil aggregate stability. Relationship between root length density (RLD) and three aggregate stability measures: slaking (a); microcracking (b); and mechanical breakdown (c) in the presence and absence of Lolium perenne. Black dots indicate communities without *L. perenne*, white dots indicate communities with *L. perenne* present.

Increased root length had a positive relationship with all measures of aggregate stability (Table 2) and although we did not directly assess the impact of specific root length in ancova model, its strong correlation with root length density may also infer a positive relationship. Root mass had a positive relationship with soil strength (*F*_1,45 _= 8.27, *P* < 0.01). To investigate whether the root traits of a community could explain the diversity effect, we fitted species richness after traits in the ancova model, and found that for aggregate stability against slaking breakdown species richness effects were still significant after fitting traits, yet the root traits explained a larger part of the variation in aggregate stability (Table 2). For the other two aggregate stability measures, species richness was not a significant factor in the models including root traits, such that root traits are likely to be an important component in the way species richness influences aggregate stability. We used further analysis to investigate how key species could be impacting on the trait‐aggregate dynamics. When *L. perenne* (the species with largest impact on aggregates and root length) was present in a community, there was no relationship between root length and aggregate stability, however, when the species was absent, the relationship was significantly positive (Fig. 2).

**Table 2:** Summary of GLM analysis of the effect of biological properties and species richness on aggregate stability in the mesocosm soils

	VIF	Soil physical properties					
		Aggregate stability					
		Resistance to slaking		Resistance to microcracking		Resistance to mechanical breakdown	
		*F*_1,51_	*P*	*F*_1,51_	*P*	*F*_1,51_	*P*
Covariates							
RLD (m/dm^3^)	1.74	**27.98**	**<0.0001 ↑**	**19.76**	**<0.0001 ↑**	**16.43**	**<0.001 ↑**
RD (g/dm^3^)	1.79	3.11	<0.1	1.631	0.21	1.51	0.23
LOI (%)	1.04	1.21	0.2774	1.09	0.30	0.06	0.80
AB (g)	1.28	0.26	0.61	0.01	0.94	1.28	0.26
Factors							
Species richness	1.27	**7.40**	**<0.01 ↑**	0.56	0.46	1.19	0.28

Displaying *F* and *P* values from ancova analysis for: the effects of root length density (RLD), root mass density (RD), soil organic matter content (LOI), aboveground biomass (AB) and the factor species richness on the three measures of aggregate stability. Species Richness was always fitted last after first fitting covariates. Text in bold indicates a significant effect (*P*<0.05) Arrows indicate a positive/negative relationship between covariate/factor and physical property. VIF indicates the variance inflation factor of each parameter.

### Field experiment

There was also a positive relationship between plant species richness and all three measures of aggregate stability in the field soils (Fig. 1d–f, Table 3). After accounting for variance attributed to community diversity, we investigated the impact of the presence of each plant functional group upon soil aggregate stability. The presence of grasses significantly increased aggregate stability (Table 3), while the presence of legumes significantly decreased all three of these stability measures (Table 3). Block effects, inferring changes in soil texture, also showed a significant relationship with all three measures of aggregate stability (Table 3). Both plant species richness and functional group richness had a positive impact on root density, glomalin‐related protein and soil organic matter content, but no effect on root length density (Supplementary Table S2). Grasses had a positive impact on root length density and mass density, while legumes had a negative effect on root length density (Supplementary Table S2). Root length density, root density and glomalin‐related protein – all had positive relationships with aggregate stability (Table 4). ancova's showed species richness still had a strong impact on slaking and mechanical breakdown resistance after first fitting root traits (Table 4).

**Table 3:** Summary of manova and anova analysis of plant community properties on soil aggregate stability in the Jena experiment soils

Factor	manova		anova			
	df	Aggregate stability	df	Aggregate stability against		
				Slaking	Microcracking	Mechanical breakdown
Block	**3.70**	5.43***	**3.70**	8.15***	4.06*	12.83***
SR first	**1.70**	26.64***↑	**1.70**	75.89***↑	27.68***↑	53.65***↑
SR second		13.13***↑		37.42***↑	8.29**↑	26.08***↑
FR first	**1.70**	14.65***↑	**1.70**	39.39***↑	22.86***↑	28.31***↑
FR second		1.14		0.92	3.47	0.75
Grass	**1.70**	9.92***↑	**1.70**	30.09***↑	5.10*↑	12.39***↑
Legume	**1.70**	9.73***↓	**1.70**	29.96***↓	7.73**↓	12.96***↓
S. Herb	**1.70**	1.38	**1.70**	0.01	1.12	1.75
T. Herb	**1.70**	0.94	**1.70**	0.1	0.17	1.14

Displaying *F* values from manova and anovas for effects of block, species richness (SR), functional group richness (FR) and the presence of grasses, legumes, short herbs and tall herbs on aggregate stability against slaking, microcracking and mechanical breakdown. SR and FR are indicated as to whether fitted before (first), or after (second) each other. Text in bold indicates a significant effect (*P < 0.05; **P < 0.01; ***P < 0.001). Arrows indicate an increase or decrease in the response as a result of the relevant factor. Species Richness log transformed prior to analysis.

**Table 4:** Results of GLM (ancova) analysis of the effects of soil biological properties and plant community factors on soil aggregate stability for the Jena experiment

	VIF	df	Soil aggregate stability		
			Slaking	Microcracking	Mechanical breakdown
Covariates					
RLD	1.69	1.65	68.48***↑	17.01***↑	38.66***↑
RD	1.71	1.65	90.08***↑	20.06***↑	86.99***↑
GRP	3.85	1.65	66.01***↑	11.98**↑	78.02***↑
LOI	3.06	1.65	111.50***↑	34.31***↑	101.76***↑
Factors					
Block	4.06	3.65	7.38***	5.99**	1.56
SR first	2.16	1.65	19.59**↑	3.70	10.22**↑
SR second	2.16	1.65	10.92**↑	0.49	5.64*↑
FR First	4.80	1.65	9.43**↑	6.39*↑	5.00*↑
FR second	4.80	1.65	0.77	3.18	0.43
Grass	3.65	1.65	5.91*↑	0.02	0.93
Legume	2.46	1.65	12.89***↓	1.60	5.70*↓

Displaying *F* values from ancovas for effects of root length density (RLD), root mass density (RD), glomalin‐related protein (GRP), organic matter (OM), block, species richness (SR), functional group richness (FR) and the presence of grasses and legumes on aggregate stability against slaking, microcracking and mechanical breakdown. Text in bold indicates a significant effect (*P < 0.05; **P < 0.01; ***P < 0.001). Arrows indicate a positive/negative correlation between covariate/factor and aggregate stability breakdown. Species Richness log transformed prior to analysis. VIF indicates the variance inflation factor of each parameter.

## Discussion

Our results show that soil aggregate stability increased under greater plant species richness and that the root traits of particular species and functional groups played a significant role in this relationship. In both the mesocosm and the long‐term field experiment soils, which differed in their physical and chemical properties, there was a significant positive influence of plant diversity on all three measures of aggregate stability, which was also paralleled by an increase in aboveground plant productivity in the mesocosms, as reported in numerous biodiversity‐ecosystem function experiments (Isbell *et al*. 2011; Cardinale *et al*. 2012). Previous studies on correlative patterns (Pohl *et al*. 2009), or less extensive diversity gradients in the field (Pérès *et al*. 2013), have detected an effect of plant species richness on aggregate stability; however, our findings, which come from a controlled mesocosm experiment and a long‐term field experiment, allowed us to investigate the relationship across a wide gradient of species richness over the longer term, and identify dominant mechanisms involved, including the role of root traits. Together, they provide strong evidence of a positive effect of plant species richness on soil aggregate stability in temperate grassland with a key role for root traits.

Roots play a major role in soil aggregation, and our results show significant links between root traits and aggregate stability. In particular, the positive effect of selected community root traits and soil biological properties on aggregate stability was found in both the mesocosm and the field experiment. In mesocosms, higher species richness increased root length and reduced the average root diameter, indicating a response in the abundance of finer roots within the soil system. The behaviour of finer rooting strategies play an integral role in developing soil structural properties (Rillig *et al*. 2015) and as such, our data suggest that higher plant diversity influences aggregate stability by increasing the contribution of finer roots to aggregate stabilisation. By testing this idea with ancova analysis, we found that root properties contributed to the apparent species richness effect for aggregate resistance to microcracking and mechanical breakdown in the mesocosms. However, root traits did not account for all variation caused by species richness for slaking in the mesocosms, or for slaking or mechanical breakdown in the field study, suggesting a diversity effect is not solely caused through pathways involving the measured belowground biological components.

Aggregate stability against microcracking, caused by gradual wetting of aggregates over time, had the least pronounced association with plant diversity in both experiments. In contrast, aggregate resistance to slaking caused by rapid wetting of aggregates was highly responsive to changes in plant diversity and showed the strongest associations with root traits and soil biological properties in both experiments. Further, the aggregate size distributions after breakdown showed slaking to be the most vigorous breakdown mechanism. These variations in response may be attributed to the mechanisms behind each breakdown. Slaking breakdown is caused by the compression of entrapped air within an aggregate, following rapid immersion in water (Le Bissonais 1996). As such, it can be controlled by the wetting rate of the aggregate and the volume of air within pore space (Le Bissonais 1996; Zaher & Caron 2008). Consequently, biological properties that can influence it include the creation, exploitation and occlusion of pore space by roots and hyphae (Caron *et al*. 1996; Six *et al*. 2004; Rillig *et al*. 2015), and the release of hydrophobic compounds that influence aggregate wetting rates (Czarnes *et al*. 2000; Hallett 2007). In particular, the degree of arbuscular mycorrhizal colonisation of plant roots, in response to shifts in diversity, could have a significant effect on soil aggregate stabilisation through hyphal extension, exudation and mortality (Rillig *et al*. 2015). Such properties were not measured here, but might have contributed to the diversity effects unaccounted for by root traits in our models. In contrast to slaking, aggregate disruption through microcracking relies on the shrink‐swell properties of the aggregate (Le Bissonais 1996), which is influenced more by intrinsic soil physical factors, rather than biological factors (Boivin *et al*. 2004); as such, microcracking is less likely to respond to changes in plant diversity and composition. Aggregate breakdown differences were also evident between the mesocosm and field experiment. In particular, soil organic matter was found to influence aggregate stability in the field, but surprisingly had no impact on aggregates in mesocosms. This effect may, in part, be due to the longer timescales and lower clay content of the field soils, which can increase the rate of organic matter accumulation (Poeplau *et al*. 2011).

Grass species had a notable effect on soil structure, benefitting aggregate stability in both the field and mesocosm experiments. In the mesocosm experiment, soils planted with monocultures of the grass *L. perenne* displayed greater aggregate stability against slaking, and the field treatments showed that irrespective of diversity treatment, the inclusion of grasses as a functional group within the mixed species plots significantly increased aggregate stability. This strong influence of grasses on soil aggregate stability may be associated with specific rooting strategies of the grasses. *L. perenne* and *A. odoratum* had the greatest root length, specific root length and narrowest average diameter of all species tested, reflecting the fine rooting behaviour of grasses, an exploitative strategy to maximise resource uptake (Hodge 2003; Cahill & McNickle 2011). Moreover, in similar mesocosm studies, the grass *A. odoratum* was found to increase its root biomass in mixed communities, particularly over the short term (Mommer *et al*. 2010, 2015). These finer rooting strategies exhibit high decomposability (Goebel *et al*. 2011; Liang *et al*. 2015; Roumet *et al*. 2016), resulting in greater organic input to soil, which in turn enhances aggregate stabilisation (Abiven *et al*. 2009). In addition, by increasing the presence of roots within the soil, aggregate stability is enhanced by enmeshment and binding from root hairs, organic exudates and the microbial stimulation associated with roots (Ritz & Young 2004). Furthermore, the grass species *L. perenne* played a substantial role in soil aggregate stabilisation; in the mesocosm communities, the inclusion of *L. perenne* significantly altered the relationship between plant community roots and soil aggregate stability. Communities in the presence of *L. perenne* had much higher soil aggregate stabilities, even when root length was low. As such, in the mesocosms, the relationship between plant species richness and aggregate stability may be attributed to the effect of *L. perenne,* and its root traits, as a component of the plant community, rather than diversity *per se*. Although the field experiment did not allow investigation of the role of single species, the inclusions of grasses in greater diversity plots may present a higher chance of including an effective soil stabiliser. As previously shown for soil biological properties (De Deyn *et al*. 2010), these findings suggest that the soil physical environment is under strong direct influence of plant species identity and composition.

In contrast to grasses, the presence of legumes was associated with lower levels of soil aggregate stability. The inclusion of legumes within the field experimental plots resulted in reduced aggregate stability, and in the mesocosms, soil planted with monocultures of the legumes *T. repens* and *L. corniculatus* generally displayed lower aggregate stability relative to grass and forb species. Legumes produced less root length than grasses, which may have limited their capacity to stabilise soil aggregates in comparison to grasses. However, despite the absence of species richness effects on soil hydraulic conductivity or strength, these measures were influenced by legumes: compared to other species, the legume *L. corniculatus* had the greatest influence on soil strength when grown in monocultures, and aboveground biomass of *L. corniculatus* was positively correlated with soil strength in mixed communities; also, soils planted with legumes displayed greater hydraulic conductivity than the other functional groups. Soil strength benefitted from increased root mass, which provides anchorage and resistance against disturbance (Waldron & Dakessian 1982; Loades *et al*. 2010). As such, the larger diameter roots and greater root mass provided by *L. corniculatus* reinforced soil strength, suggesting the inclusion in grassland communities of certain species with influential root reinforcing traits, such as *L. corniculatus*, could play a substantial role in increasing slope stability. Although legumes are known to influence key nutrient cycling processes (Spehn *et al*. 2002; Milcu *et al*. 2008), these findings demonstrate their capacity to also have beneficial effects on soil physical structure.

Our results show that high plant species diversity and particular suites of root traits benefit a range of soil physical properties in grassland that are key to soil functioning. Plant species richness was found to have strong positive effects on soil aggregate stability and the presence of certain species of grasses and legumes, and their root traits, was found to strongly influence soil physical properties of grassland in contrasting and potentially complementary ways. Relationships between plant identity and soil physical properties were strongly influenced by root traits. Fine rooting grass species, and in particular, *L. perenne,* had a strong relationship with soil aggregate stability, while the presence of legumes, especially *L. corniculatus* with its thicker root systems*,* resulted in less stable soil aggregates, but benefited soil hydrologic and strength properties. As such, our results suggest potential for a combination of these species within a community to display a range of complementary effects upon soil physical properties. Globally, soil physical degradation presents a serious threat to the provisioning of ecosystem services and human well‐being (Amundson *et al*. 2015; FAO 2015; Keesstra 2016). The significant links between plant community diversity and composition and soil physical properties we reveal may provide the basis for combating soil physical degradation and restoring soil function, allowing the maintenance and restoration of ecosystem services.

## Authorship

RDB and JNQ secured funding for this study. The mesocosm experiment was initiated and designed by IJG, RDB and JNQ, using an original experimental design of RDB and GBDD. The field experiment was conducted by IJG, with assistance from AW and GBDD. Laboratory work was undertaken by IJG, AW and GBDD, and IJG performed all statistical analysis. IJG wrote the manuscript with contributions from all authors. The underlying data in this paper is available from DOI 10.17635/lancaster/researchdata/86.
