# Supplementary material for: Plant diversity and root traits benefit physical properties key to soil function in grasslands
Source: Ecol Lett. 2016 Jul 26;19(9):1140–9. doi: 10.1111/ele.12652 (PMC4988498; doi:10.1111/ele.12652)
Supplement: Supplementary file 1 [file ELE-19-1140-s001.docx]

**Supplementary Methods**

The mesocosms were saturated for 48 hours before Ksat measurements were taken. The falling head method of measuring Ksat was employed, using the entire mesocosm pot as a permeameter. Additional narrow piping was attached and sealed to the top, thus any fall in head can be read accurately in the narrower-diameter duct. Using Darcy’s principles of saturated hydraulic conductivity, we measured the rate of flow of water based on the area and length of the soil column, and the fall in head over time. Ksat was calculated with Equation (1): Ksat= (A1 x L)/(A2 x t)ln(h0/h1), where A1 is the cross sectional area of the standpipe and A2 is the cross sectional area of the mesocosm soil column (L represents the length of the soil column, t is the time period and h0 and h1 are head heights at t=0 and t=t) (Supplementary Figure 1). For each mesocosm, t was recorded after a drop in head height of 10 mm, and repeat measures were taken on each mesocosm until two concurrent readings were recorded. After Ksat measurements, the mesocosm pots were re-saturated for 24 hours. Immediately prior to strength testing, a pre-determined shear plane at 8cm soil depth was exposed by removal of the pot lining. We developed a shearing rig that fitted complimentary to each mesocosm pot (see supplementary methods development), which applied a shear load to the exposed shear plane. A hydraulic ram was used to apply constant loading to the soil column, whilst force and displacement of the top half of the soil column were recorded with a Campbell Scientific CR800 datalogger. For each individual mesocosm, we created a force-displacement graph to derive a value of root reinforcement of shear strength for each mesocosm by subtracting the peak force measured from each treatment with the average peak force measured from the bare soil pots. After strength measurements, root mass density only was determined.

**Supplementary Methods Development: Root reinforcement of soil shear strength**

Having reviewed both conventional, and adapted, laboratory shearboxes for measuring shear strength of root-permeated soil, we established specifications for a custom-made direct shear testing device for measuring the influence of plant diversity on the root reinforcement of soil in a mesocosm experiment. A design for a direct shear testing rig was developed, and manufactured, by A. E. Huddleston Ltd, Quernmore.

Our shear testing rig comprised a metal frame made compatible to fit around 64 pots that contain the experimental soil. The rig primarily consisted of two components: a static base that fits around the pot, and a moveable top section with the capacity to slide horizontally along two runners. A hydraulic jack was affixed to the rear of this top section, which provided the horizontal shear force to test soil strength. The jack was connected to a hydraulic pump, which gave a constant horizontal loading force throughout shearing. This horizontal movement forces the top half of soil, above the exposed shear plane, to deform for a distance of 200 mm, allowing readings of force and displacement to be logged during the shearing process. Force was recorded with an STA-4 tension/compression load cell (LCM Systems) fixed to the end of the jack, whilst horizontal displacement was recorded with a 200 mm PD-13 displacement transducer (LCM systems) attached to the side of the rig. Data from both load cell and displacement transducer were collated through a CR800 data logger (Campbell Scientific). The calibration factor for the displacement transducer was: y = 19.64 – 𝘹/298, where 𝘹 is the transducer output, and y is the total displacement in mm. The calibration factor for the load cell was: y = 25.129𝘹, where 𝘹 is the cell output, and y is the total force recorded in kN/m^2^. The entire rig had adjustable and removable legs, so that shear plane height settings could be adjusted for any future use beyond this project.

In order to get successful force and displacement readings from the shearing process, the experimental soil had to be in a container (mesocosm) compatible with the shear testing rig. For this experiment, we used a 300 mm diameter 4 mm thick twin walled piping to create mesocosm pots. Within each pot, we made a pre-determined shear plane by cutting through the pipe at 8 cm soil depth, in effect creating two separate pipe halves to the pot, one above and one below the shear plane, which were fixed back together with reinforced adhesive tape. Just prior to shear testing, this tape could then be removed exposing a shear plane of soil. As we were carrying out the shearing on saturated soils, we also lined the inside of each pot with a watertight polyethene layer, which could be cut at the level of the shear plane just prior to testing. Once each pot was made suitable with the pre-determined shear plane and lining, 35 cm of soil was uniformly packed in at a density of 1.3 g/cm^3^ at 7cm intervals above a 10 cm layer of drainage gravel, after which seedlings were planted at a uniform density in the top of the pot. After 18 months of root growth, each mesocosm pot was then ready for the shearing process.

48 hours prior to shear testing, each pot was saturated from the bottom up, in order to expel any air pockets upwards, and create saturated conditions for strength testing. After the wetting period, the outer adhesive tape, and inner lining, around the pre-determined shear plane was cut. This exposed a plane of soil now reinforced with roots. The shear testing rig was then placed over each pot, aligning with the shear plane. Once the rig was fixed in place, the hydraulic pump was operated allowing the hydraulic jack to horizontally displace the upper layer of soil above the shear plane. Force and displacement measurements were recorded in real time in ASCII files on the CR800 data logger.

Force and displacement ASCII files recorded from the logger were then uploaded to Microsoft Excel, where force-displacement graphs could be generated for each sample sheared. For comparison between different planted soils in the mesocosm experiment, we interpreted the peak shearing force for each treatment, and subtracted from this the mean value for peak shearing force from the unplanted bare soil pots, allowing us an indicator for the contribution of roots to soil strength.


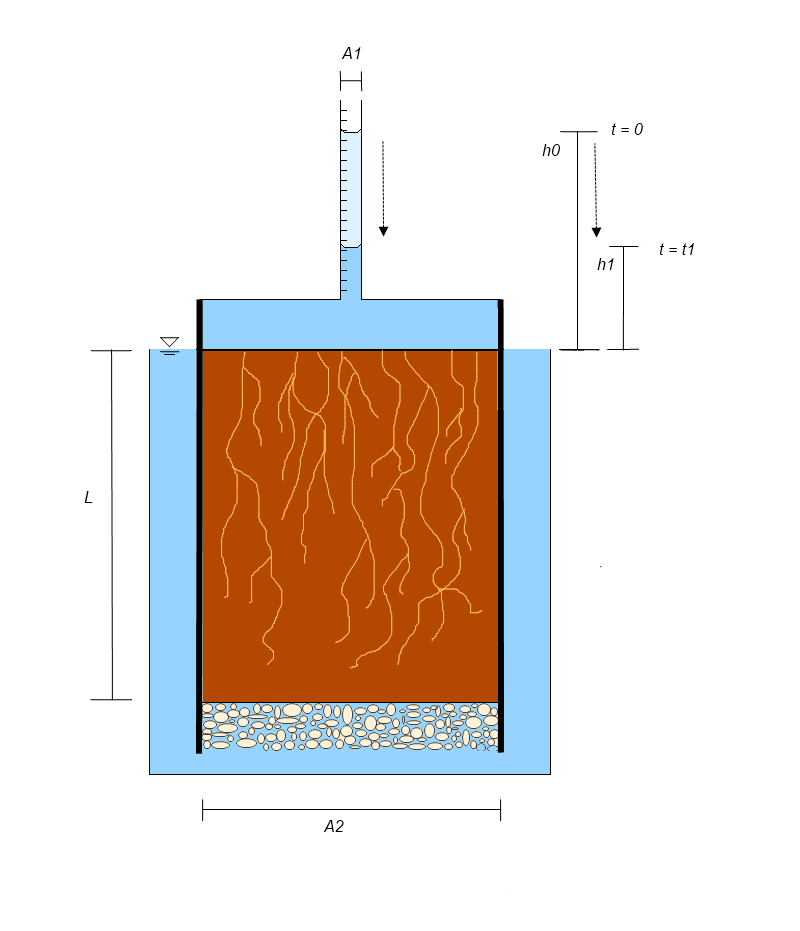


**Supplementary Figure 1: Diagram of mesocosm set up for measurement of hydraulic conductivity.**
