# Supplementary material for: Plant diversity and root traits benefit physical properties key to soil function in grasslands
Source: Ecol Lett. 2016 Jul 26;19(9):1140–9. doi: 10.1111/ele.12652 (PMC4988498; doi:10.1111/ele.12652)
Supplement: Supplementary file 2 [file ELE-19-1140-s002.docx]

**Supplementary Table 1:** Summary of GLM analysis of species richness on soil properties in the mesocosm soils.

| Soil Properties |  |  |  |  |  | |  | |
| --- | --- | --- | --- | --- | --- | --- | --- | --- |
|  |  |  |  |  |  | |  | |
|  | *df* |  |  | F | p | |  | |
| **Physical Properties** |  |  |  |  |  | |  | |
| Slaking | 1,55 |  |  | **15.65** | **<0.01** ↑ | | | |
| Microcracking | 1,55 |  |  | **4.09** | **<0.05**↑ | | | |
| Mechanical breakdown | 1,55 |  |  | **6.69** | **<0.05**↑ | | | |
| Saturated hydraulic conductivity | 1,55 |  |  | 3.75 | | 0.06 | | |
| Root reinforcement of soil strength | 1,45 |  |  | 0.05 | | 0.82 | | |
|  |  |  |  |  | |  | |  |
| **Biological Properties** |  |  |  |  | |  | |  |
| RLD | 1,55 |  |  | **7.19** | | **<0.01↑** | |  |
| RD | 1,55 |  |  | 1.6 | | 0.21 | |  |
| RDIAM | 1,55 |  |  | **5.21** | | **<0.05↓** | |  |
| SRL | 1,55 |  |  | 2.50 | | 0.11 | |  |
| DMC | 1,55 |  |  | **4.81** | | **<0.05↓** | |  |
| TMD | 1,55 |  |  | 0.06 | | 0.81 | |  |
| LOI | 1,55 |  |  | 0.00 | | 0.96 | |  |
| AB | 1,55 |  |  | **6.17** | | **<0.05↑** | |  |
| Displaying F and p values for GLM analysis of the effect of species richness on soil physical properties, root length density (RLD), root mass density (RD), root diameter (RDIAM), specific root length (SRL), dry matter content (DMC), tissue mass density (TMD), organic matter content (LOI) and aboveground biomass (AB). Where significant (bold) arrows indicate positive or negative effect. | | | | | | | | |
|  |  |  |  |  |  |  |  |  |
|  |  |  |  |  |  |  |  |  |
|  |  |  |  |  |  |  |  |  |
