# Supplementary material for: Plant diversity and root traits benefit physical properties key to soil function in grasslands
Source: Ecol Lett. 2016 Jul 26;19(9):1140–9. doi: 10.1111/ele.12652 (PMC4988498; doi:10.1111/ele.12652)
Supplement: Supplementary file 3 [file ELE-19-1140-s003.docx]

Supplementary Table 2: Effects of Species richness and community composition on soil biological and physical properties in the Jena (field) soils

|  |  | | Soil biological properties | | |  |  | Aggregate Stability | | |  |
| --- | --- | --- | --- | --- | --- | --- | --- | --- | --- | --- | --- |
| Factor | df | | RLD | RD | GRP | OM |  | Slaking | Microcracking | | Mechanical |
|  | |  |  |  |  |  |  |  |  | |  |
| Block | | 3,70 | 2.04 | 0.84 | **37.43***** | **21.18***** |  | **8.15***** | **4.06*** | | **12.83***** |
|  | |  |  |  |  |  |  |  |  | |  |
| SR first | | 1,70 | 0.91 | **14.64*****↑ | **17.36*****↑ | **32.35*****↑ |  | **75.89*****↑ | **27.68*****↑ | | **53.65*****↑ |
| SR second | | | 0.57 | **6.31***↑ | **10.90****↑ | **16.99*****↑ |  | **37.42*****↑ | **8.29****↑ | | **26.08*****↑ |
| FR First | | 1,70 | 0.34 | **8.81****↑ | **6.47***↑ | **15.54*****↑ |  | **39.39*****↑ | **22.86*****↑ | | **28.31*****↑ |
| FR second | | | 0 | 0.49 | 0.02 | 0.18 |  | 0.92 | 3.47∙↑ | | 0.75 |
|  | |  |  |  |  |  |  |  |  | |  |
| Grass | | 1,70 | **34.98*****↑ | **12.36*****↑ | 0.74 | 0.67 |  | **30.09*****↑ | **5.10***↑ | | **12.39*****↑ |
| Legume | | 1,70 | **14.36*****↓ | 2.22 | 0.97 | 2.5 |  | **29.96*****↓ | **7.73****↓ | | **12.96*****↓ |
| S. Herb | 1,70 | | 0.17 | 0.97 | 0.15 | 3.81∙↑ |  | 0.01 | 1.12 | | 1.75 |
| T. Herb | 1,70 | | 2.17 | 0.87 | 0.05 | 1.22 |  | 0.1 | 0.17 | | 1.14 |
| Displaying *F* values from ANOVAs for effects of block, species richness (SR), functional group richness (FR), and the | | | | | | | | | | | |
| presence of Grasses, Legumes, Short herbs and Tall herbs on root length density (RLD), root mass density (RD), | | | | | | | | | | | |
| glomalin-related protein (GRP), organic matter (OM), and aggregate stability against slaking, microcracking and | | | | | | | | | | | |
| mechanical breakdown. SR and FR are indicated as to whether fitted before, or after each other. Text in bold | | | | | | | | | | | |
| indicates a significant effect to p> 0.05. Arrows indicate an increase or decrease in the response as a result of the | | | | | | | | | | | |
| relevant factor. | | |  |  |  |  |  |  |  |  | |
| * p <0.05; ** p<0.01; *** p<0.001 | | | | |  |  |  |  |  |  | |
