# Supplementary material for: Plant diversity and root traits benefit physical properties key to soil function in grasslands
Source: Ecol Lett. 2016 Jul 26;19(9):1140–9. doi: 10.1111/ele.12652 (PMC4988498; doi:10.1111/ele.12652)

**Supplementary Graphs: Aggregate breakdown distribution under different plant species monocultures**

Slaking


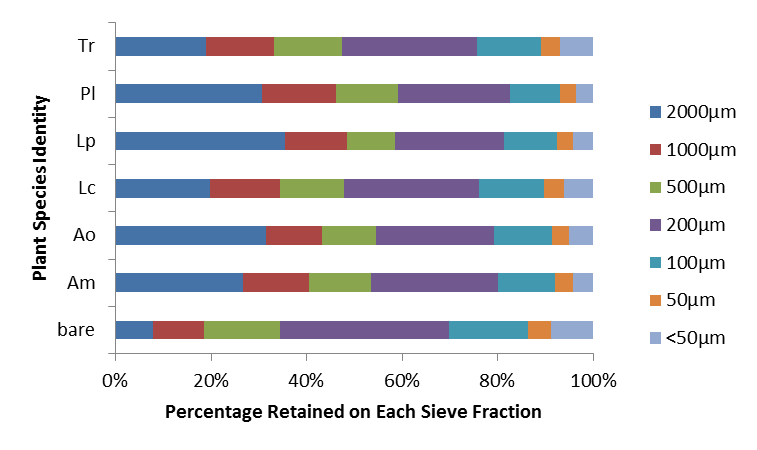


Microcracking


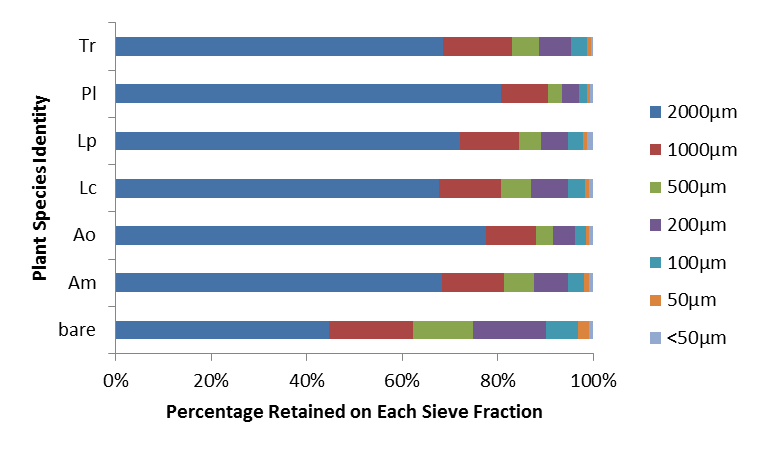


Mechanical Breakdown


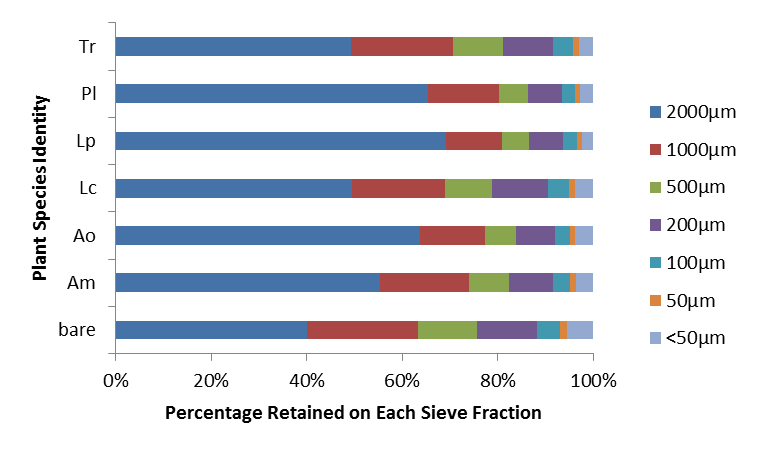


**Supplementary Graphs: Aggregate breakdown distribution under diversity treatment in mescosms**

Slaking


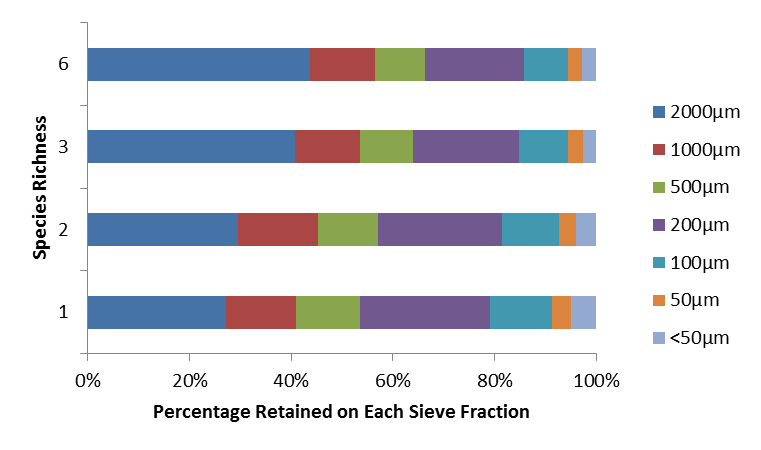


Microcracking


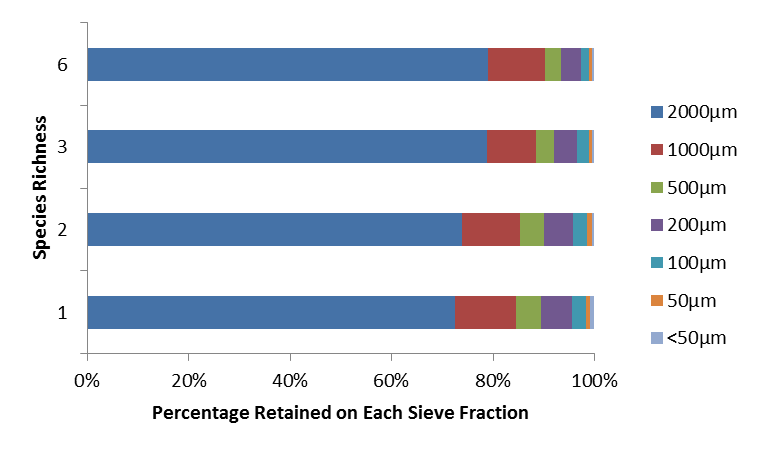


Mechanical Breakdown


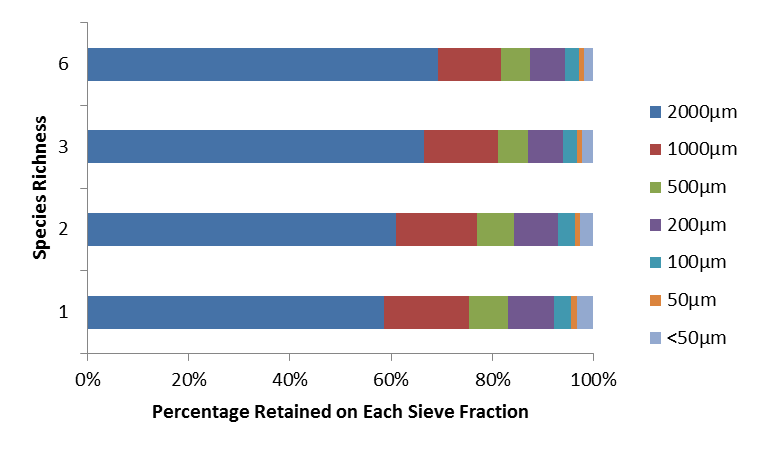


**Supplementary Graphs: Aggregate breakdown distribution under diversity treatment in the field experiment**

Slaking


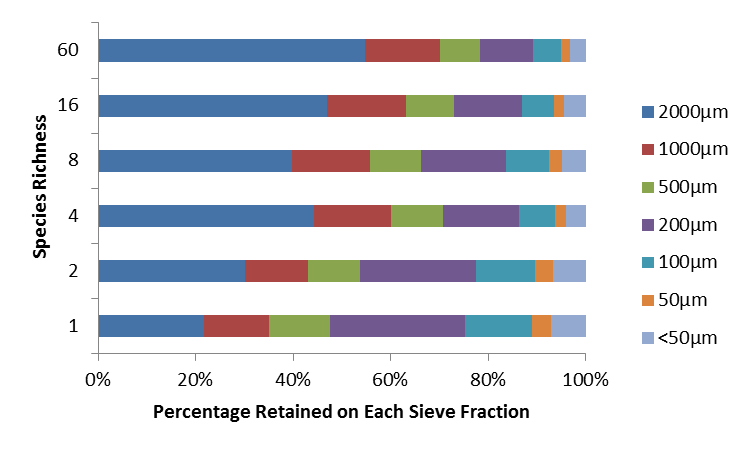


Microcracking


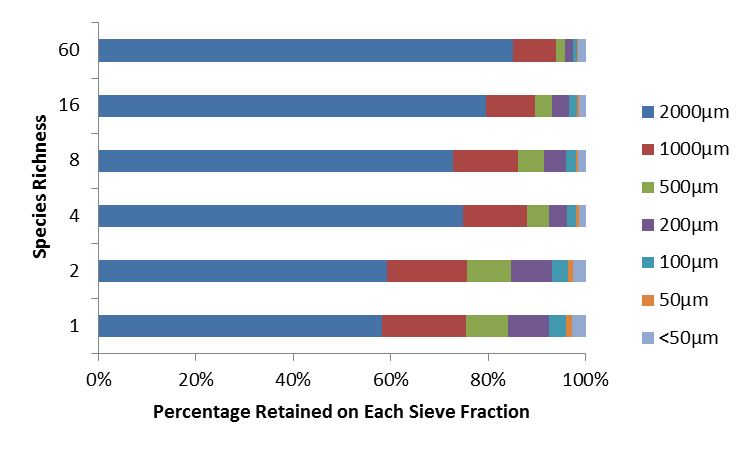


Mechanical Breakdown


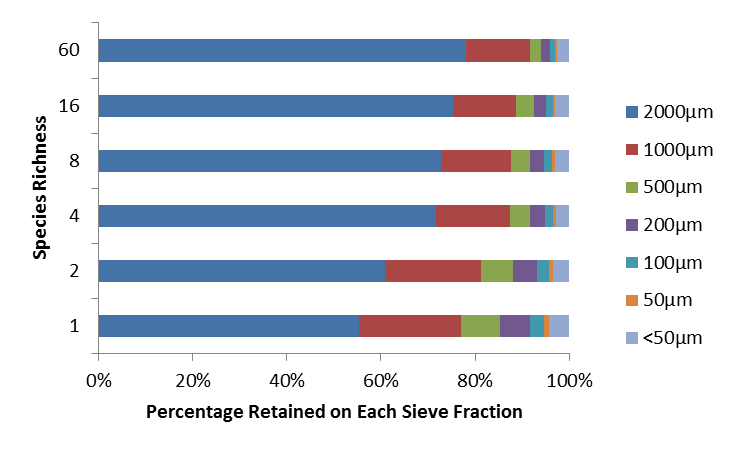

Supplement: Supplementary file 4 [file ELE-19-1140-s004.docx]
